# Novel AChE Inhibitors for Sustainable Insecticide Resistance Management

**DOI:** 10.1371/journal.pone.0047125

**Published:** 2012-10-08

**Authors:** Haoues Alout, Pierrick Labbé, Arnaud Berthomieu, Luc Djogbénou, Jean-Paul Leonetti, Philippe Fort, Mylène Weill

**Affiliations:** 1 Universités Montpellier 2 et 1, Montpellier, France; 2 CNRS UMR 5554, Institut des Sciences de l'Evolution, Montpellier, France; 3 Institut Régional de Santé Publique/Université d'Abomey-Calavi, Cotonou, Benin; 4 CNRS UMR 5236, Centre d'étude d'agents Pathogènes et Biotechnologies pour la Santé, Montpellier, France; 5 CNRS UMR 5237, Centre de Recherche en Biochimie Macromoléculaire, Montpellier, France; National Institute for Communicable Diseases/NHLS, South Africa

## Abstract

Resistance to insecticides has become a critical issue in pest management and it is particularly chronic in the control of human disease vectors. The gravity of this situation is being exacerbated since there has not been a new insecticide class produced for over twenty years. Reasoned strategies have been developed to limit resistance spread but have proven difficult to implement in the field. Here we propose a new conceptual strategy based on inhibitors that preferentially target mosquitoes already resistant to a currently used insecticide. Application of such inhibitors in rotation with the insecticide against which resistance has been selected initially is expected to restore vector control efficacy and reduce the odds of neo-resistance. We validated this strategy by screening for inhibitors of the G119S mutated acetylcholinesterase-1 (AChE1), which mediates insensitivity to the widely used organophosphates (OP) and carbamates (CX) insecticides. PyrimidineTrione Furan-substituted (PTF) compounds came out as best hits, acting biochemically as reversible and competitive inhibitors of mosquito AChE1 and preferentially inhibiting the mutated form, insensitive to OP and CX. PTF application in bioassays preferentially killed OP-resistant *Culex pipiens* and *Anopheles gambiae* larvae as a consequence of AChE1 inhibition. Modeling the evolution of frequencies of wild type and OP-insensitive AChE1 alleles in PTF-treated populations using the selectivity parameters estimated from bioassays predicts a rapid rise in the wild type allele frequency. This study identifies the first compound class that preferentially targets OP-resistant mosquitoes, thus restoring OP-susceptibility, which validates a new prospect of sustainable insecticide resistance management.

## Introduction

Organophosphates (OP), carbamates (CX) and pyrethroids represent, by number, 80% of insecticides used in the field (reviewed in [1]). These molecules act on the nervous system through inhibition of acetylcholinesterase (OP and CX) or voltage-gated sodium channels (pyrethroids and DDT). The major setback of insecticide use is the selection for resistance, observed not only in the targeted pests but also in many other sympatric species [2], [3]. At the physiological level, resistance is a consequence of either increased detoxication or modification of the insecticide target, the latter often resulting in very high insensitivity [4], [5]. However both mechanisms may be responsible for vector control failure and have to be addressed by insecticide resistance management strategies. Resistance has spread to such an extent, particularly in mosquito vector populations, that it now represents a critical issue for the control of the diseases they transmit, e.g. malaria, dengue, filariasis, West Nile fever or Japanese encephalitis [6].

Sustainable strategies to counter resistance spread aim at maintaining resistant alleles at frequencies low enough so that current insecticides remain efficient even at moderate doses. As an example, the reasoned use of insecticides through rotations or mosaic applications takes advantage of the pleiotropic cost (i.e. the reduced fitness of resistant vs. wild type individuals in an insecticide-free environment) to maintain resistant alleles at low frequencies (reviewed in [7]). Essentially used for malaria control, fungi also represent promising tools because they kill mosquitoes at slower rate than insecticides thus reducing the risk of resistance selection [8], [9], [10]. Here we propose an alternative approach based on the development of "resistant killer" compounds, capable of preferentially inhibiting targets already insensitive to a given insecticide class. Combined with the fitness cost already associated with resistance, populations treated with such "resistant killers" are thus expected to regain a high frequency of susceptible wild type alleles, a "hit where it already hurts" strategy. Ideally, the targeted protein should be highly constrained structurally to minimize its capacity to evolve through the selection of new mutations that would confer resistance to both the insecticide and the "resistant killer" compound.

A good candidate is acetylcholinesterase (AChE, EC 3.1.1.7), which in Coelomates acts as a synaptic terminator of nerve impulses through hydrolysis of the neurotransmitter acetylcholine. Mosquitoes contain two AChE genes (*ace-1* and *ace-2*), *ace-1* encoding the synaptic enzyme [11], [12]. So far, only three substitutions on residues lining the catalytic site confer OP and CX insensitivity to AChE1: the F331W substitution (amino-acid numbering according to the *Torpedo californica* AChE nomenclature [14]), found only in *Culex tritaeniorhynchus* [13], [14], the F290V substitution, found only in *C. pipiens* species [13], and the universally found G119S substitution, which confers the highest level of insensitivity to a broad range of insecticides and was selected independently in several *Culex* and *Anopheles* species (*C. pipiens pipiens*, *C. pipiens quinquefasciatus*, *C. vishnui*, *A. gambiae* and *A. albimanus*, [15], [16], [17], [18]). The G119S-substituted AChE1 appeared as a suitable candidate for the development of reverser compounds because AChE are highly structurally constrained [19] and the G119S mutation is widely distributed worldwide in mosquitoes and associated with a substantial fitness cost in insecticide-free areas [20], [21], [22], [23], [24]. This AChE1 constraint constitutes a weakness in its adaptive capacity that might be used to develop innovative resistance management strategies: an insecticide targeting specifically the G119S AChE1 should efficiently reduce the frequency of the resistance allele, while the probability of OP-resistant mosquitoes developing a secondary resistance to the new insecticide is predicted to be quite low. To address the feasibility of this approach, compounds were screened for their capacity to inhibit more efficiently the G119S-substituted (OP-insensitive) AChE1 than the wild type (WT) AChE1. Further biochemical analysis, bioassays on mosquito larvae from susceptible and resistant *A. gambiae* and *C. pipiens* strains sharing the same genetic background, as well as gene population modeling show that application of compounds with such properties is predicted to rapidly restore OP susceptibility in field populations.

## Results

### Identification of Inhibitors of the OP-insensitive G119S AChE1

**Figure 1:**
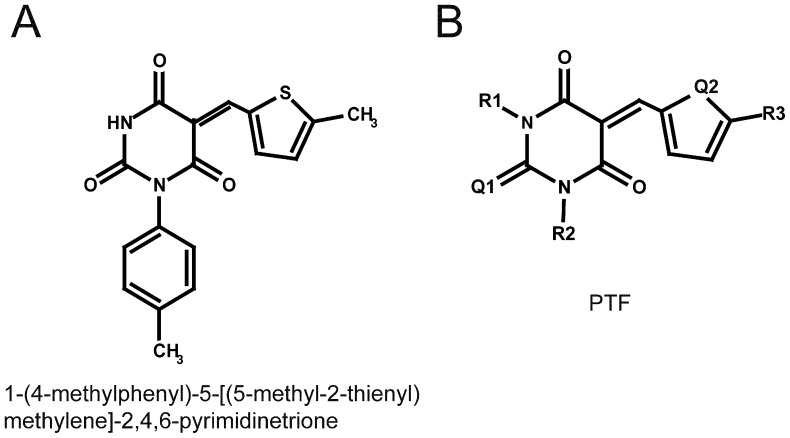
Structures of the hit compound (A) and active analogs (B). R_1_ is a hydrogen atom or a methyl group, R_2_, a methyl group or a substituted aryl group, R_3_, mostly oxygen or sulfur atoms, except for two compounds having a N atom or a substituted nitrogen group. Q_1_ is oxygen or sulfur atoms and Q_2_ is a variable chemical group.

To identify inhibitors of G119S AChE1, we screened a 3,000-compound chemical library using an assay adapted to a microplate format [25], [26]. Nine compounds reduced the apparent hydrolysis of the acetylthiocholine substrate, of which one gave an 80–87% inhibition. The core structure of this compound is made of a heterocyclic 2,4,6-pyrimidinetrione (barbituric acid) substituted by a furan cycle (Figure 1A). To improve the efficacy, 71 commercial analogs sharing the core structure with various substitutions on R_1_, R_2_, R_3,_ Q_1_ and Q_2_ positions were assayed (Figure 1B). For simplification, these compounds will be termed from now as PTF (PyrimidineTrione Furan-substituted) and were classified depending on the substituted positions (Table S1). PTFs exhibited a broad IC_50_ range on WT and G119S AChE1 (Table S1), from 0.23 µM to 4.5 mM. When sorted by their relative efficacy toward the G119S AChE1 (Table 1), 31 analogs had a R_IC50_ (ratio of the mean IC_50_ on WT over the mean IC_50_ on G119S AChE1) above that of the hit compound (PTF-24, R_IC50_ = 0.7). Seven analogs had a R_IC50_ above 10, indicating a much higher efficacy toward G119S AChE1 than OP-sensitive AChE1. Six among the most efficient and specific PTFs (in bold, Table 1) also showed preferential inhibition on other types of OP-insensitive AChE1 (F290V and F331W substitutions) (Table S2). Last, PTFs behaved as reversible and competitive inhibitors; Loss of inhibition after dilution of the enzyme-inhibitor complex indicates that PTFs do not bind covalently to AChE1 (Figure S1); Plotting 1/*v* (Dixon plots) and [S]/*v* (Cornish-Bowden plots) against inhibitor concentration (Figure S2) indicates that inhibition is competitive since for various substrate concentrations, the enzyme maximal velocity V_max_ did not vary (panels A and B) and [S]/*v* lines were parallel (panels C and D). Comparison of dissociation constants of the enzyme inhibitor complexes (Kic) confirmed the preferential binding to the OP-insensitive AChE1 (Table S3).

**Table 1:** Biochemical and larvicidal properties of the most potent PTFs.

PTF^a^	Group^a^	IC_50_ (µM)^b^			Mortality at 300 µM^c^		
		WT	G119S	R_IC50_ b	Slab	SR	R_m300_ c
33^f^	B	490.0	22.0	22.3	16%	77%	4.8
52	C	860.0	52.0	16.5	0%	0%	−
8^f^	A	312.0	19.0	16.4	60%	100%	1.7
**25** d,f	**B**	**442.0**	**28.6**	**15.5**	**32%**	**68%**	**2.1**
37	B	2121.0	142.0	14.9	0%	35%	−
7	A	424.0	35.0	12.1	100%	100%	1.0
**20** d,f	**A**	**330.0**	**27.5**	**12.0**	**43%**	**94%**	**2.2**
**39** d,f	**C**	**74.3**	**7.6**	**9.8**	**29%**	**81%**	**2.8**
69	F	43.9	4.6	9.5	0%	0%	−
12	A	54.0	6.0	9.0	46%	88%	1.9
4	A	111.0	14.0	7.9	78%	100%	1.3
**10** d,f	**A**	**592.0**	**81.4**	**7.3**	**14%**	**87%**	**6.2**
16	A	260.0	37.0	7.0	80%	100%	1.3
60	D	7.7	1.1	6.8	50%	50%	1.0
9^f^	A	132.0	21.5	6.1	19%	39%	2.1
**3** d,f	**A,D**	**29.4**	**6.2**	**4.7**	**43%**	**78%**	**1.8**
**29** d,f	**B**	**118.0**	**33.5**	**3.5**	**27%**	**63%**	**2.3**
38	C	1189.0	357.0	3.3	70%	90%	1.3
45	C	3.4	1.1	3.1	0%	0%	−
18	A	5.3	1.8	2.9	0%	0%	−
70	F	18.0	6.2	2.9	0%	0%	−
13^f^	A	236.0	89.0	2.7	64%	96%	1.5
56	D	4.9	1.9	2.6	0%	0%	−
59	D	45.7	18.5	2.5	0%	0%	−
47	C	3.0	1.3	2.3	0%	0%	−
2^f^	A	12.6	5.5	2.3	12%	53%	4.4
30	B	2.8	1.3	2.2	25%	25%	1.0
57	D	11.3	5.4	2.1	0%	0%	−
66^f^	F	91.6	51.0	1.8	63%	100%	1.6
35	B	8.7	8.7	1.0	5%	49%	9.8
23	B	12.1	15.5	0.8	24%	60%	2.5
24^e^	B	1.3	1.9	0.7	0%	0%	−
17	A	5.0	8.0	0.6	15%	90%	6.0

a numbers and groups refer to Table S1.

b IC_50_ values were determined from regression analysis of log-concentrations versus percentage inhibitions. R_IC50_ =  IC_50_ WT/IC_50_ G119S. Compounds were sorted by their R_IC50_ ratio.

c Mortality was measured from bioassays on Slab (OP-sensitive) and SR (OP-insensitive) strains exposed for 24 hours to 300 µM PTF. R_m300_ =  SR mortality/Slab mortality.

d PTFs biochemically characterized are in bold.

e Hit compound from the primary screen.

f Compounds with R_IC50_ and R_m300_ above 1.5.

#### PTF insecticide activity on OP-susceptible and resistant strains

**Figure 2:**
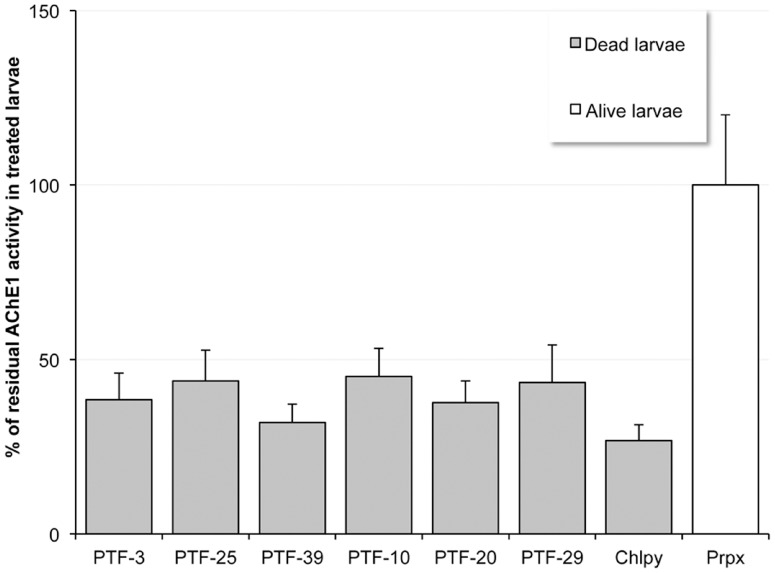
Relative AChE1 activity in OP-resistant SR larvae killed by PTF treatment. *C. pipiens* larvae from the OP-resistant SR strain were exposed to 300 µM PTF until death and then AChE1 residual activity was measured. Chlorpyrifos (Chlpy) and propoxur (Prpx) were used as positive and negative control inhibitors, respectively. Means and standard errors for three independent experiments are shown.

The 71 analogs were screened for their toxicity on OP-susceptible (Slab strain) or OP-resistant (SR stain) *C. pipiens* larvae. As a first approach, each PTF was applied at 300 µM for 24 hours (Table S1). Sixteen PTFs were at least 50% more efficient on OP-resistant larvae (ratio R_m300_ of mortality of SR larvae over mortality of Slab larvae above 1.5 in Table 1), among which thirteen were also selective as biochemical inhibitors (R_IC50_ ratios above 1.5, Table 1). We ensured that the mortality induced by PTFs was associated with AChE1 inhibition by measuring the residual AChE1 activity in killed *C. pipiens* SR larvae (Figure 2). Exposure to PTFs elicited a 55 to 70% reduction in AChE1 activity, within the range of that elicited by exposure to chlorpyrifos (75%), at a dose where it kills OP-resistant larvae through AChE1 inhibition. This demonstrates that PTF larvicidal activity is a consequence of *in vivo* AChE1 inhibition. To investigate further the larvicidal activities of PTFs, bioassays were performed on OP-resistant and OP-susceptible *C. pipiens* and *A. gambiae* larvae (Table 2). PTF-3, -10, -20, -25, -29 and -39 had LD_50_ ranging from 70.1 to 398.8 µM on OP-resistant *C. pipiens* larvae (SR strain) and from 160.7 to 964 µM on susceptible ones (Slab strain), with R_LD50_ (ratio of the mean LD_50_ on susceptible strain over the mean LD_50_ on resistant strain) ranging from 1.5 to 3.9. *Anopheles gambiae* had a similar pattern of susceptibility: PTFs preferentially killed OP-resistant larvae (Acerkis strain), with R_LD50_ values ranging from 1.3 to 7.7. Each PTF showed species-specific toxicity, but to our knowledge, PTFs are the only molecules that display a higher toxicity on OP-resistant G119S strains than on susceptible strains, both in *C. pipiens* and in *A. gambiae*.

To determine the dominance of the larvicidal activity of PTFs against *ace-1* alleles, six PTFs were tested on heterozygous (*ace-1^R^/ace-1^S^*) larvae produced by a cross between Slab and SR strains, and also on the *C. pipiens* Ducos strain, harboring the duplicated *ace-1* allele in which one *ace-1^S^* and one *ace-1^R^* copy are arranged in tandem at the homozygous state [27]). Although the Ducos strain showed slightly less susceptibility, all PTFs tested showed a similar efficacy on [R/S] heterozygotes phenotypes compared to [R/R] homozygotes larvae, indicating dominance of the OP-insensitive *ace-1^R^* allele (Table 2).

**Table 2:** PTF toxicity (LD_50_) on *C. pipiens* and *A. gambiae* larvae from OP-susceptible and OP-resistant strains.

	LD_50_ a						R_LD50_ b		
PTF	Slab^c^	SR^c^	Ducos^c^	F_1_ (SR×Slab)	Kisumu^d^	Acerkis^d^	Slab/SR	Slab/Ducos	Acerkis
3	310.0	143.0	162.1	151.3	109.7	86.6	2.17	1.90	1.27
	(238.8–512.3)	(109.8–192.1)	(140.3–188.8)	(135.1–169.6)	(100.3–121.9)	(76.2–98.3)			
10	300.0	206.0	232.8	207.1	388.3	50.6	1.46	1.30	7.67
	(227.9–608.3)	(162.8–280.7)	(186.8–320.2)	(175.4–254.1)	(278.5–748.9)	(29.0–71.4)			
20	762.0	194.0	NA e	NA e	NA e	NA e	3.93	−	−
	(305.7–1.1×10^7^)	(132.2–405.2)							
25	160.8	70.1	130.8	60.0	46.1	12.6	2.29	1.20	3.64
	(130.2–208.3)	(60.6–79.7)	(114.3–150.3)	(53.3–67.5)	(14.4–73.0)	(0.0–32.4)			
29	946.0	398.8	409.0	105.1	164.4	67.7	2.37	2.30	2.43
	(517.2–4.3×10^7^)	(315.4–616.3)	(301.8–722.3)	(91.9–119.6)	(137.1–200.3)	(41.1–93.8)			
39	576.0	200.0	201.9	110.0	NA e	NA e	2.88	2.90	−
	(350.1–3.7×10^6^)	(156.8–261.6)	(174.2–236.7)	(97.3–124.3)					
Propoxur	1.6	2.1×10^3^	56.7	2.3	0.1	689.6	7.5×10^−4^	2.8×10^−2^	1.9×10^−4^
	(0.2–13.4)	(1.9–2.7×10^3^)	(18.2–205.5)	(5.8×10^−2^–9.4×10^3^)	(0.08–0.36)	(577.3–781.4)			
Chlorpyrifos	1.9×10^−4^	2.1×10^−2^	2.2×10^−3^	1.5×10^−3^	9.5×10^−4^	0.6	9×10^−3^	8.6×10^−2^	1.6×10^−3^
	(0.6–8.9)×10^−4^	(0.6–7.2) ×10^−2^	(1.9–2.5) ×10^−3^	(0.8–2.2) ×10^−3^	(0.7–3.3) ×10^−3^	(2.9–0.2)			

a Four to five replicates were performed for each bioassay. LD_50_ is expressed in µM. 95% confidence intervals are indicated into parentheses.

b Ratio of LD_50_ for OP-susceptible to LD_50_ for OP-resistant strains.

c *Culex pipiens* strains: Slab, OP-susceptible *ace-1^S/S^*; SR, OP-resistant *ace-1^R/R^*; Ducos, OP-resistant *ace-1^D^* (duplication).

d *Anopheles gambiae* strains: Kisumu, OP-susceptible *ace-1^S/S^*; Acerkis, OP-resistant *ace-1^R/R^.*

e NA: not analyzed.

#### PTFs as predicted to restore OP-susceptibility

To examine what would be the impact of PTFs or compounds of similar properties on the evolution of the frequency of resistant alleles in natural populations, we ran a simulation in which the initial infinite and panmictic population contained 10% of OP-sensitive allele (*ace-1* ^S^), a situation already observed in several OP-treated areas [28], [29], [30], and in which there was no migration. PTF doses were fixed at LD_50_ for SR larvae and used a *r* ratio (mortality of resistant *ace-1* ^R^ over that of susceptible *ace-1* ^S^ homozygotes) ranging from 1.2 to 6.0 (*i.e.* within the range of R_m300_ or R_LD50_ measured for the six PTFs in Tables 1 and 2), and up to 100. We also examined the extreme situations where the *ace-1^R^* allele is recessive (*i.e*. heterozygotes have the same susceptibility to the inhibitor as *ace-1^S^* homozygotes) or dominant (*ace-1^R^* dominant, *i.e*. heterozygotes have the same susceptibility to the inhibitor as *ace-1^R^* homozygotes).

**Figure 3:**
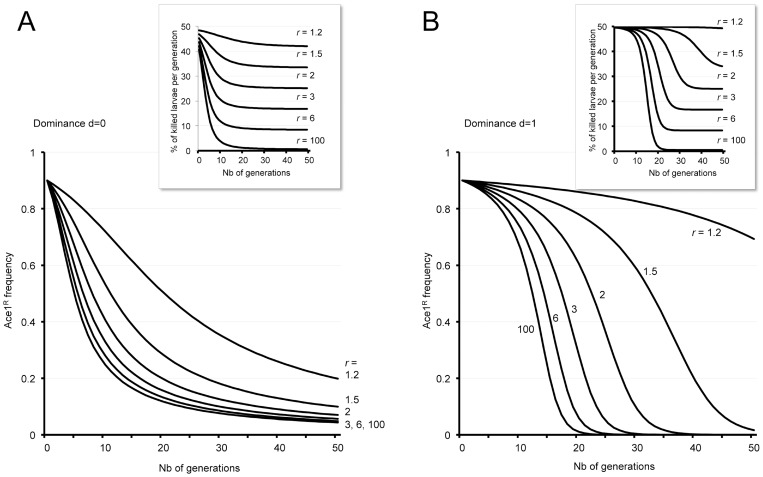
Modeling the impact of PTF treatment on frequency of the *ace-1^R^* allele and on larvae survival. Evolution of an OP-resistant, infinite and panmictic population treated with PTFs was computed as described in Material and Methods. The *ace-1^R^* initial frequency was 0.9 and PTF compounds were applied at LD_50_ for *ace-1^R^* homozygotes (i.e. *m*  = 0.5). *r* represents the mortality ratio of *ace-1^R^* vs. *ace-1^S^* homozygotes. Panel **A** represents the evolution of *ace-1^R^* frequency when this allele is recessive (*d*  = 0) and panel **B,** when it is dominant (*d*  = 1). Curves represent the evolution of *ace-1^R^* frequency across generations for various PTF mortality *r* ratios between 1.2 and 100. Insets represent the proportion of individuals killed at each generation.

Under the recessive model (*d*  = 0, Figure 3A), *ace-1^R^* frequency (*p*) decreases rapidly even at *r*  = 1.2 (*p*  = 0.4 after 27 generations). Increasing *r* up to 6 accelerates the process (*p*  = 0.4 after only 10 generations), while higher values do not improve it significantly further: the *ace-1^R^* allele is indeed rapidly restricted to heterozygotes, weakly susceptible to inhibitors with high *r*-values. Under this scenario, the OP-resistant allele remains in the population, albeit at low frequency, even after 50 generations. Under the dominant model (*d*  = 1, Figure 3B), *ace-1^R^* frequency *p* is more sensitive to *r*, as it is not silent in heterozygotes: *p* decreases at a slow rate for *r* below 1.5 and at a much faster rate for *r* above 2. As for recessivity, increasing *r* beyond 6 has minor effects on the decrease rate of *ace-1^R^*, but in contrast, *ace-1^R^* is eliminated from the population in less than 50 generations if *r* ≥1.5. As shown in the insets of panels A and B, the percentage of individuals killed at each generation (i.e. the efficacy of vector control) is only significantly reduced for toxicity ratios *r* above 2. This occurs after 10 to 30 generations depending on the dominance type, since global mortality during the first generations is mostly driven by the initial *ace-1^R^* frequency (*p_0_* = 0.9) and the mortality rate of *ace-1^R^* homozygotes (*m*  = 0.5, i.e. at LD_50_ dose).

In conclusion, this model shows that treating populations with PTF compounds allows to regain OP-susceptibility; PTF application to populations with high OP-resistance allele frequency should lead in a few generations to both significant mortality and decrease in OP-resistant alleles frequency.

## Discussion

We show here that PTFs qualify as a new class of reversible and competitive AChE1 inhibitors, with preferential efficacy toward OP-insensitive AChE1 *in vitro* and OP-resistant *C. pipiens* or *A. gambiae* mosquito larvae *in vivo*.

The fact that PTFs behave as reversible and competitive AChE1 inhibitors strongly suggests that they target the docking sites for acetylcholine, *i.e*. the peripheral (P-) or the active (A-) sites [31]. Furthermore, PTFs preferentially inhibit all OP-insensitive mutants (G119S, F290V, F331W), whose substituted positions line the A-site and are thought to confer OP-insensitivity by steric hindrance [17], [31]. It is therefore unlikely that PTFs can freely enter the A-site of OP-insensitive enzymes and a more likely hypothesis is that PTFs bind on top of the A- or the P-site, thereby blocking access to the substrate to either site. This agrees with the absence of correlation between PTF efficacy and specific chemical groups at the R_1_, R_2_ or R_3_ positions, suggesting that docking of the core PTF structure to its target might be stabilized through multiple interactions with neighboring residues. Availability of AChE1 three-dimensional structure, alone or in complex with PTFs, should help understand the structural basis of the interaction.

PTFs proved to be efficient *in vivo* on *C. pipiens* and *A. gambiae* larvae and showed similar selectivity toward OP-resistant larvae, although with different R_LD50_ values. Behavioral or physiological differences might account for this difference; *Anopheles gambiae* larvae are surface filter-feeders while *C. pipiens* are deeper filter-feeders that regularly swim up to breathe [32]. Previous work also demonstrated that the dominance of resistance in *C. pipiens* varies with environment, such as food, water quality and shape of the cups used in bioassays [33]. All these parameters might impact on insecticide uptake or induced mortality.

Since PTFs inhibit all types of OP-insensitive AChE1 *in vitro*, i.e. G119S-, F290V- or F331W-substituted, this strongly suggests that PTF molecules might exhibit a broad larvicidal spectrum toward most OP-resistant field populations. Availability of PTFs as a new insecticide class directed against OP-insensitive AChE1 represents a major advance for the development of sustainable insecticide resistance management strategies for three main reasons:

- PTF-like compounds are expected to efficiently control populations with high OP-resistant allele frequencies while reducing the risk of selecting resistance alleles other than the wild type. Indeed, AChE1 is a structurally highly constrained protein and the G119S substitution conferring OP-resistance has substantially reduced its enzymatic activity [34]. Occurrence of an additional substitution on the OP-insensitive backbone is thus likely to further decrease AChE1 activity below the required physiological levels.
- Modeling the impact of PTFs on an OP-resistant population indicates that even moderate selectivity (*r* = 1.5 to 3) is sufficient to significantly decrease the frequency of OP-resistant alleles (i.e. effective resistance control) in just a few generations. PTF-treated populations might therefore rapidly regain OP-susceptibility and be subsequently controlled by OPs at much lower doses. Used in rotation, PTFs and OPs would thus be complementary tools for controlling mosquito populations and managing resistance in a more sustainable way.
- Compounds that target OP-sensitive and insensitive AChE1 might also prove very effective on populations that have selected for *ace-1* duplication, associating a wild type and a resistant G119S allele, like in *C. pipiens* and *A. gambiae* populations heavily controlled by insecticides [25], [28], [35]. This duplication was shown to partially compensate for the fitness cost associated with mutated AChE1, probably by restoring cholinergic activity close to physiological levels [27]. Interestingly, the *ace-1^R^* allele appeared mostly dominant toward PTF in a context where both G119S and WT AChE1 forms are present [i.e. Ducos strain (*ace-1^D^*) or (*ace-1^R^/ace-1^S^*) heterozygotes, Table 2]. According to the model predictions, this further supports the use of PTFs for rapidly decreasing the frequency of *ace-1^R^* in OP-resistant populations.

In conclusion, this study validates an innovative approach for resistance management of mosquito vectors, based on the development of molecules targeting preferentially enzymes already insensitive to currently used insecticides. PTFs identified here behave as preferential inhibitors of AChE1 mutants insensitive to OP and CX and as preferential killers of OP-resistant *C. pipiens* and *A. gambiae* larvae in bioassays. This approach should allow both efficient vector and resistance control management: The preferential killing of resistant mosquitoes mimics a situation of negative cross-resistance, in which frequency of resistance alleles decreases much faster than when insecticides acting on other targets are used. Furthermore, since wild type OP-susceptible alleles are, by construction, PTF-resistant alleles, odds are against the selection of other resistance events. Wild type alleles are indeed more frequent than any spontaneous mutant and are associated with the highest fitness. Although not easily amenable to high throughput screening, voltage-gated sodium channels represent other interesting candidates for developing a similar strategy; they are targets of pyrethroids, the major insecticide class currently used in malaria control; resistance to pyrethroids is spreading in many species through the selection of a very small number of insensitive alleles (particularly *kdr-west, kdr-east*), affecting the same amino acid 1014 (house fly numbering) [36], [37]. This strategy could also be applied to any pest that acquired resistance through one or a few mutations in a structurally constrained target for which resistance is associated with a fitness cost.

Developing new approaches to maintain vector control and maximize the effective lifespan of current and future insecticides is one of the objectives of the Global Plan for Insecticide Resistance Management (WHO 2012). This aim is paramount in a context where more than 500 arthropod species (either medical or agricultural pests) have become resistant to most if not all currently used insecticides [38]. The present study demonstrates that a "hit where it already hurts" strategy could fit the bill.

## Materials and Methods

### Chemicals

All chemical compounds were purchased from ChemBridge (San Diego, CA, USA), except eserine, from Sigma-Aldrich, (Saint-Louis, USA), propoxur, from Bayer (Leverkusen, Germany), chlorpyrifos, from CIL Luzeau (France) and tacrine, from ICN Biomedicals, Inc (Eschwege, Germany).

### Strains

Three *C. pipiens* strains were used: the susceptible reference Slab strain, homozygous for *ace-1* ^S^ [39], the resistant reference SR strain, carrying the genetical background of Slab but homozygous for the G119S mutation, allele *ace-1* ^R^ [21] and the Ducos strain, carrying the genetical background of Slab but homozygous for an *ace-1* duplication, with one *ace-1^S^* and *ace-1^R^* copy in tandem [27]. *Culex pipiens* heterozygous larvae (Slab×SR) were obtained by crossing Slab males and SR females. Two *A. gambiae* reference strains (S molecular form) were used: the susceptible strain Kisumu, homozygous for *ace-1* ^S^, collected in Kenya in 1953 and maintained since then under laboratory conditions [40] and the resistant Acerkis strain, carrying the genetical background of Kisumu but homozygous for an *ace-1^R^* allele from a population collected in Bobo-Dioulasso, Burkina Faso [41].

### AChE Assays and Screening Procedures

Production of *C. pipiens* WT and mutated AChE1s in *Drosophila* S_2_ cells was already described [17]. The chemical library (ChemBridge, 3,000 compounds) screening was performed in duplicate on G119S recombinant AChE1. Chemicals were made soluble in ethanol or dimethylsulphoxide (DMSO), and then diluted at 300 µM in ethanol for storage. Compounds (30 µM final concentration) were incubated for 15 min at room temperature with 100 µl of G119S recombinant AChE1 then 100 µl of substrate (acetylthiocholine, 1.6 mM, Sigma-Aldrich) was added and the residual activity was quantified by measuring the optical density at 412 nm, as described by Ellman et al. [26]. PTF analogs were analyzed in dose-response experiments (10-fold serial dilutions from 3 mM to 30 nM), using recombinant WT and G119S AChE1 or mosquito head extracts (heads cut from frozen mosquitoes, homogenized in 400 µl PB containing 1% Triton X-100 and cleared by centrifugation at 9,000×g for 3 min). Depending on compound availability, two to five replicates were performed with distinct batches of enzyme. Concentrations producing 50% enzyme inhibition (IC_50_) were determined using regression analysis of log-concentrations *versus* percentage inhibitions. IC_50_ was estimated by nonlinear least square regression. IC_50_ was also measured on AChE1 from susceptible and resistant mosquito larvae extracts.

To address the residual AChE1 activity after exposure to 300 µM PTF, larvae were collected as soon as mortality was reached to avoid AChE1 degradation. Larvae were rinsed twice with distilled water and homogenized individually in 400 µl PB containing 1% Triton X-100. Homogenates were centrifuged at 9,000×g for 3 min and assayed for AChE1 activity as described above.

### Toxicological Assays

Insecticidal activity was determined by performing bioassays on fourth-instar larvae as described in [42]. Each compound was dissolved in ethanol. Propoxur (CX) and chlorpyrifos (OP) were used as references. For each compound, we first tested mortality after a 24-hour exposure at 300 µM. For the most selective compounds and depending on their availability, three to five replicates at four different concentrations were performed on *C. pipiens* and *A. gambiae* strains. Mortality data were analyzed by the log-probit program [43]. This program takes into account natural mortality and provides lethal doses (LD) and slopes for each mortality line; it also computes resistance ratios (RR) for each LD, with 95% confidence intervals.

### Modeling PTF Treatment of OP-resistant Populations

The evolution at each generation *i* of *p_i_,* the frequency of the OP-resistant allele (named *ace-1^R^*), and *N_i_,* the proportion of the population killed by insecticide, were modeled in an infinite population under panmixia (Hardy-Weinberg equilibrium). Let *m* be the mortality rate for the *ace-1^R^* homozygotes and *m*/*r* the mortality for the *ace-1^S^* homozygotes, *r* being the toxicity ratio specific for each PTF (i.e. the ratio of the mortality of *ace-1^R^* homozygotes over the mortality of *ace-1^S^* homozygotes for a given dose of a given PTF). Let *d* be the dominance of *ace-1^R^* over *ace-1^S^* in heterozygotes treated with PTF: *d* = 1 and *d* = 0 indicate, respectively, that the mortality of the heterozygotes is equal to that of the *ace-1^R^* homozygotes or to that of the *ace-1^S^* homozygotes; dominance is intermediate when 0< *d* <1. At generation *i*, PTF treatment kills a proportion.
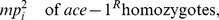


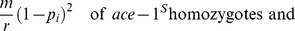


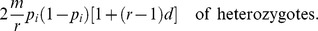



At generation *i+1*, this leads to:
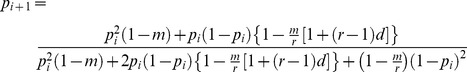
And



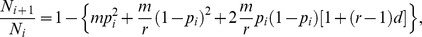
where 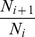 is the proportion of surviving mosquitoes between two generations. The recursions were computed and plotted using the Microsoft Office Excel software.

## Supporting Information

Figure S1 **Reversibility of PTF inhibition.** Reversibility was tested using a rapid dilution procedure. Residual activity of WT (white bars) and G119S (grey bars) AChE1 were measured after 15 min incubation in presence of inhibitors (striped bars) and are expressed as percentages of control activity. Tacrine and chlorpyrifos-oxon were used as references for reversible and irreversible inhibition, respectively. Inhibitor concentrations were 150 µM of PTF-29 for both WT and G119S AChE1, 5 and 100 µM of chlorpyrifos-oxon respectively for WT and G119S AChE1, and 5 and 50 µM tacrine respectively for WT and G119S AChE1. Assays were then diluted ten times and residual activity was measured (open bars). Means and standard errors for three independent experiments are shown.
(PDF)

Figure S2 **PTF inhibition is competitive.** WT and G119S recombinant AChE1s were incubated with 0.25 mM, 0.5 mM or 1 mM inhibitor. Residual activity of G119S (A and C) and WT (B and D) AChE1 was measured in the presence of various concentrations of PTF-20 and substrates. Enzymatic activity was analyzed using the graphical method developed by Dixon [44], representing reciprocal rates (1/*v*) (A and B) or reciprocal rates multiplied by substrate concentrations ([S]/*v*) (C and D) as a function of inhibitor concentration.
(PDF)

Table S1 **Properties of PTF analogs.**
(PDF)

Table S2 **Activities of PTFs on G119S, F290V and F331W OP-insensitive AChE1.**
(PDF)

Table S3 **Dissociation constant (K** ***ic*** **) of the enzyme-inhibitor complex.**
(PDF)
