# Supplementary figures and images for: Novel AChE Inhibitors for Sustainable Insecticide Resistance Management
Source: PLoS One. 2012 Oct 8;7(10):e47125. doi: 10.1371/journal.pone.0047125 (PMC3466212; doi:10.1371/journal.pone.0047125)

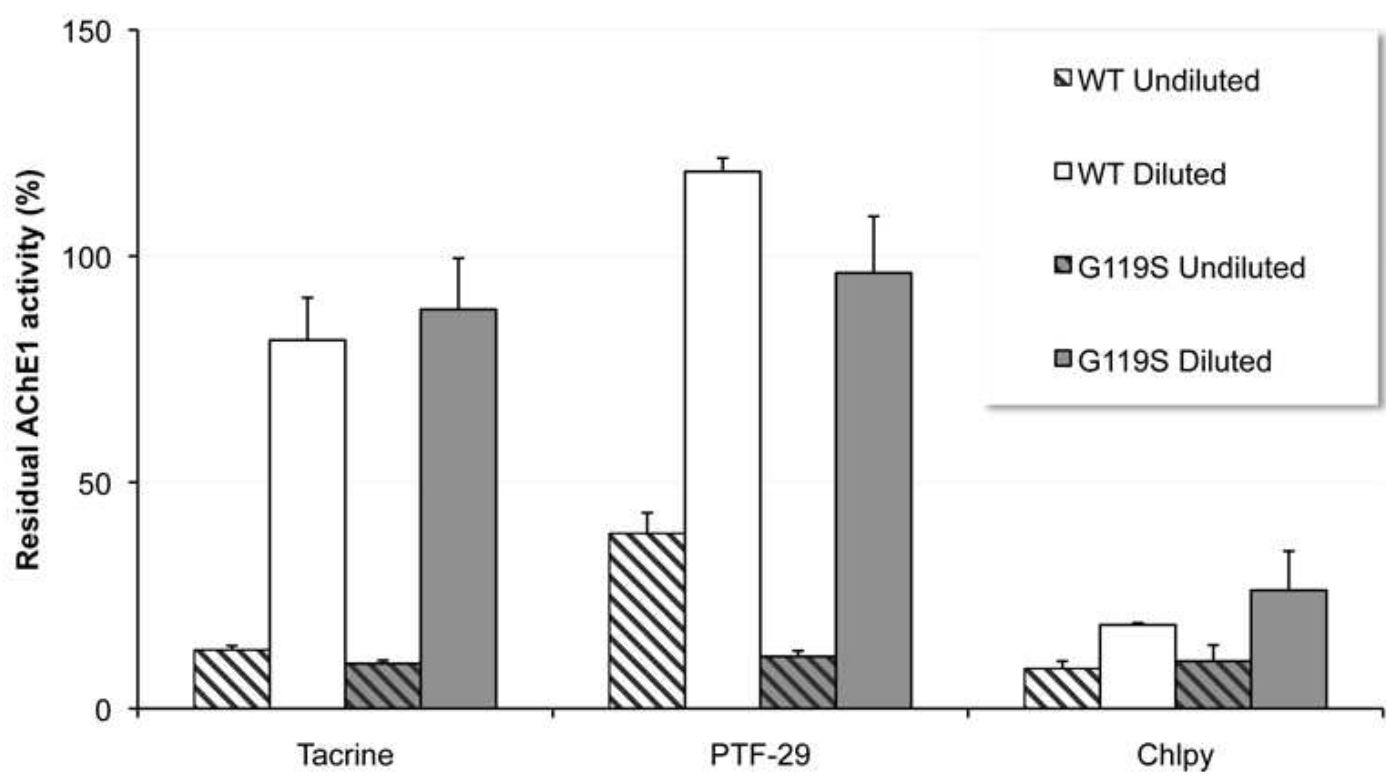

Supplement: Figure S1 — Reversibility of PTF inhibition. Reversibility was tested using a rapid dilution procedure. Residual activity of WT (white bars) and G119S (grey bars) AChE1 were measured after 15 min incubation in presence of inhibitors (striped bars) and are expressed as percentages of control activity. Tacrine and chlorpyrifos-oxon were used as references for reversible and irreversible inhibition, respectively. Inhibitor concentrations were 150 µM of PTF-29 for both WT and G119S AChE1, 5 and 100 µM of chlorpyrifos-oxon respectively for WT and G119S AChE1, and 5 and 50 µM tacrine respectively for WT and G119S AChE1. Assays were then diluted ten times and residual activity was measured (open bars). Means and standard errors for three independent experiments are shown. (PDF) [file pone.0047125.s001.pdf]

G119S AChE1

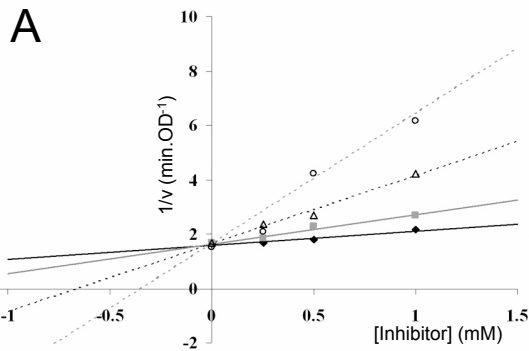

wt AChE1

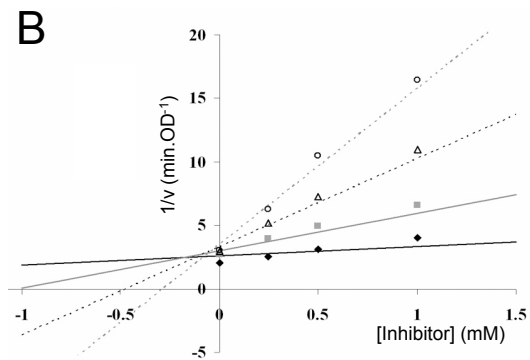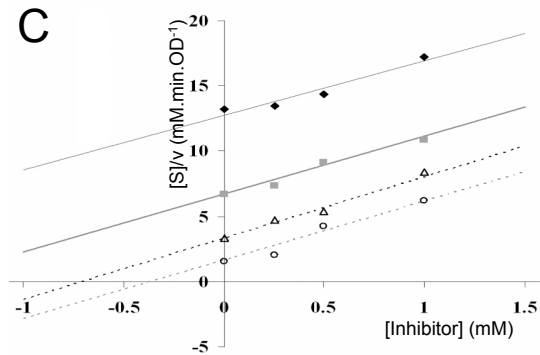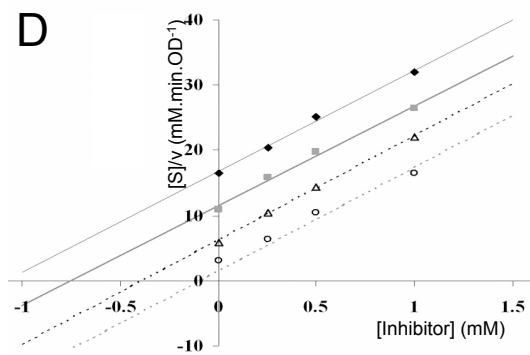

Supplement: Figure S2 — PTF inhibition is competitive. WT and G119S recombinant AChE1s were incubated with 0.25 mM, 0.5 mM or 1 mM inhibitor. Residual activity of G119S (A and C) and WT (B and D) AChE1 was measured in the presence of various concentrations of PTF-20 and substrates. Enzymatic activity was analyzed using the graphical method developed by Dixon [44], representing reciprocal rates (1/v) (A and B) or reciprocal rates multiplied by substrate concentrations ([S]/v) (C and D) as a function of inhibitor concentration. (PDF) [file pone.0047125.s002.pdf]
