# Supplementary material for: Novel AChE Inhibitors for Sustainable Insecticide Resistance Management
Source: PLoS One. 2012 Oct 8;7(10):e47125. doi: 10.1371/journal.pone.0047125 (PMC3466212; doi:10.1371/journal.pone.0047125)
Supplement: Table S1 — Properties of PTF analogs. (PDF) [file pone.0047125.s003.pdf]

Table S1. Properties of PTF analogs.

R_1- R_3 and Q_1- Q_2 are positions substituted as indicated in Figure 1. Groups A, B and F gather compounds that differ only in R_2, groups C and D, only in R_3, group E, only in Q_1. Group F gathers compounds that differ from other groups in Q_2. IC_{50} values were determined on recombinant wild-type (WT) and OP-insensitive (G119S) *C. pipiens* ACHE1 as described in Material and Methods. The R_{IC50} ratio indicates the specificity towards G119S AChE1 (IC_{50}WT/ IC_{50}G119S). Mortality was measured from bioassays on Slab (OP-sensitive) and SR (OP-insensitive) *C. pipiens* strains treated for 24 hours with 300 mM PTF. Two to five replicates were performed and standard errors (all below 10%) were removed for clarity. The R_{m300} ratio (SR mortality to Slab mortality) represents the specificity of PTFs at 300 mM towards the SR strain. Data from tacrine, propoxur, chlorpyrifos-oxon, paraoxon and dichlorvos are shown as control AChE inhibitors.

Groupe A

PTF-	Chembridge	Chemical group (position)					IC_{50} (µM)			Mortality at 300 µM		
N°	ID	R_1	R_2	R_3	Q_1	Q_2	WT	G119S	R_{IC50}	Slab	SR	R_{m300}
1	6624438	H	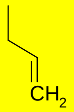	CH_3	O	O	3.0	2.2	1.4	0%	0%	-
2	6527192	H	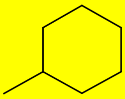	CH_3	O	O	12.6	5.5	2.3	12%	53%	4.4
3	5809912	H	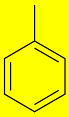	CH_3	O	O	29.4	6.2	5.0	43%	78%	1.8
4	5810115	H	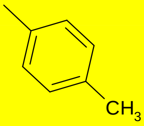	CH_3	O	O	111.0	14.0	8.0	78%	100%	1.3
5	5810021	H	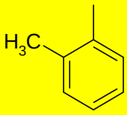	CH_3	O	O	1.4	1.1	1.3	0%	0%	-
6	5810152	H	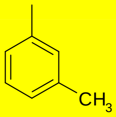	CH_3	O	O	4.0	4.4	0.9	0%	0%	-
7	6357191	H	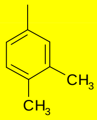	CH_3	O	O	424.0	35.0	12.0	100%	100%	1.0
8	7044564	H	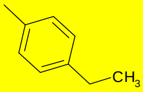	CH_3	O	O	312.0	19.0	16.0	60%	100%	1.7
9	5960869	H	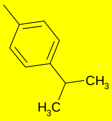	CH_3	O	O	132.0	21.5	6.0	19%	39%	2.1
10	5809948	H	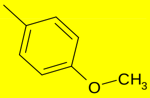	CH_3	O	O	592.0	81.4	7.0	14%	87%	6.2

11	5809984	H	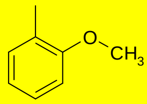	CH_3	O	O	1.3	1.0	1.4	0%	0%	-
12	5810198	H	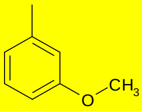	CH_3	O	O	54.0	6.0	9.0	46%	88%	1.9
13	5993354	H	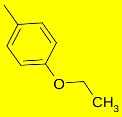	CH_3	O	O	236.0	89.0	3.0	64%	96%	1.5
14	6237811	H	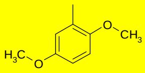	CH_3	O	O	2.0	2.0	1.0	0%	0%	-
15	6434558	H	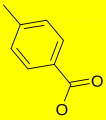	CH_3	O	O	9.0	12.9	0.7	0%	0%	-
16	6369764	H	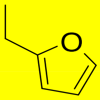	CH_3	O	O	260.0	37.0	7.0	80%	100%	1.3
17	8068010	H	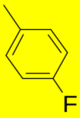	CH_3	O	O	5.0	8.0	0.6	15%	90%	6.0
18	6627875	H	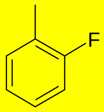	CH_3	O	O	5.3	1.8	2.9	0%	0%	-
19	6632183	H	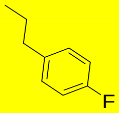	CH_3	O	O	3.3	3.7	0.9	0%	0%	-
20	5810058	H	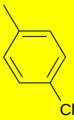	CH_3	O	O	330.0	27.5	12.0	43%	94%	2.2
21	7367799	H	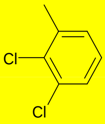	CH_3	O	O	5.0	4.8	1.0	0%	0%	-
22	5997811	H	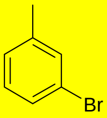	CH_3	O	O	1.5	1.9	0.8	68%	97%	1.4

Groupe B

PTF-	Chembridge	Chemical group (position)					IC_{50} (µM)			Mortality at 300 µM		
N°	ID	R_1	R_2	R_3	Q_1	Q_2	WT	G119S	R_{IC50}	Slab	SR	R_{m300}
23	7113388	H	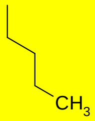	CH_3	S	O	12.1	15.5	0.8	24%	60%	2.5
24	6014971	H	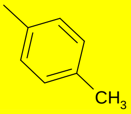	CH_3	S	O	1.3	1.9	0.7	0%	0%	-
25	5810154	H	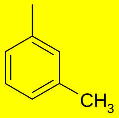	CH_3	S	O	442.0	28.6	15.0	32%	68%	2.1
26	6631273	H	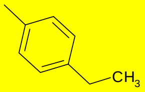	CH_3	S	O	9.0	16.0	0.6	100%	65%	0.7
27	5809951	H	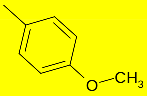	CH_3	S	O	1.7	5.9	0.2	12%	15%	1.3
28	5809987	H	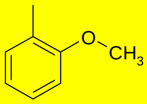	CH_3	S	O	2.1	1.8	1.1	0%	0%	-
29	5810201	H	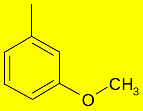	CH_3	S	O	118.0	33.5	4.0	27%	63%	2.3
30	6629858	H	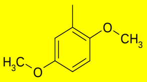	CH_3	S	O	2.8	1.3	2.2	25%	25%	1.0
31	6431063	H	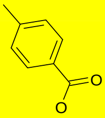	CH_3	S	O	0.5	0.2	2	20%	20%	1.0
32	6942871	H	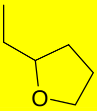	CH_3	S	O	2.1	2.3	0.9.0	0%	0%	-
33	6316393	H	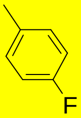	CH_3	S	O	490.0	22.0	22.0	16%	77%	4.8
34	7118489	H	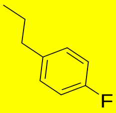	CH_3	S	O	24.0	38.0	0.6	0%	0%	-
35	6028908	H	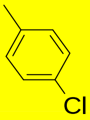	CH_3	S	O	8.7	8.7	1.0	5%	49%	9.8
36	6240216	H	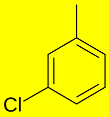	CH_3	S	O	11.8	8.0	1.5	30%	40%	1.3
37	6001277	H	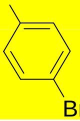	CH_3	S	O	2121.0	142.0	15.0	0%	35%	-

Groupe C

PTF-	Chembridge	Chemical group (position)					IC_{50} (µM)			Mortality at 300 µM		
N°	ID	R_1	R_2	R_3	Q_1	Q_2	WT	G119S	R_{IC50}	Slab	SR	R_{m300}
38	5174670	CH_3	CH_3	H	O	O	1189.0	357.0	3.3	70%	90%	1.3
39	5850181	CH_3	CH_3	CH_3	O	O	74.3	7.5	10.0	29%	81%	2.8
40	8045830	CH_3	CH_3	NO2-	O	O	83.0	55.0	1.5	0%	0%	-
41	6569943	CH_3	CH_3	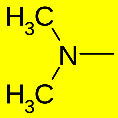	O	O	175.0	132.0	1.3	20%	20%	1.0
42	6433806	CH_3	CH_3	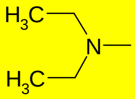	O	O	720.0	1035.0	0.7	0%	0%	-
43	5376777	CH_3	CH_3	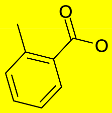	O	O	6.7	8.3	0.8	0%	0%	-
44	5377448	CH_3	CH_3	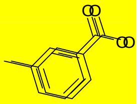	O	O	0.6	0.6	1.1	0%	0%	-
45	6522729	CH_3	CH_3	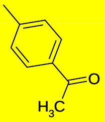	O	O	3.4	1.1	3.1	0%	0%	-
46	5527784	CH_3	CH_3	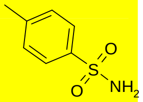	O	O	1.7	5.2	0.3	0%	0%	-
47	6064049	CH_3	CH_3	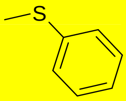	O	O	3.0	1.3	2.3	0%	0%	-
48	6318867	CH_3	CH_3	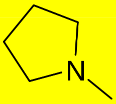	O	O	755.0	1525.0	0.5	0%	0%	-
49	6320092	CH_3	CH_3	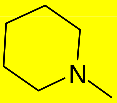	O	O	97.0	65.5	1.5	13%	0%	0
50	6510665	CH_3	CH_3	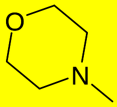	O	O	260.0	145.0	1.8	0%	0%	-

51	6422017	CH_3	CH_3	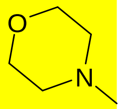	O	O	413.5	1500.0	0.3	0%	0%	-
52	6584174	CH_3	CH_3	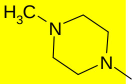	O	O	860.0	52.0	17	0%	0%	-
53	7095782	CH_3	CH_3	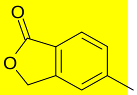	O	O	0.6	5.6	0.1	0%	0%	-

Groupe D

PTF- N°	Chembridge ID	Chemical group (position) R$_1$	R$_2$	R$_3$	Q$_1$	Q$_2$	IC$_{50}$ (µM) WT	G119S	R$_{IC50}$	Mortality at 300 µM Slab	SR	R$_{m300}$
3	5809912	H	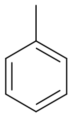	CH_3	O	O	29.4	6.2	5.0	43%	78%	1.8
54	5174640	H	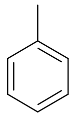	H	O	O	1394.0	737.0	1.9	100%	100%	1.0
55	5963881	H	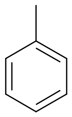	Br	O	O	17.0	32.6	0.5	0%	0%	-
56	5968871	H	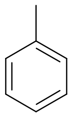	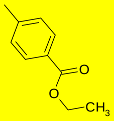	O	O	4.9	1.9	2.6	0%	0%	-
57	5981137	H	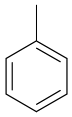	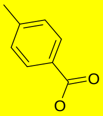	O	O	11.3	5.4	2.1	0%	0%	-
58	5988008	H	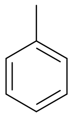	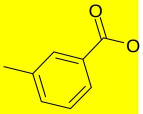	O	O	6.7	9.3	0.7	0%	0%	-
59	6026239	H	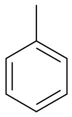	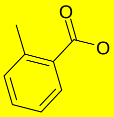	O	O	45.7	18.5	2.5	0%	0%	-
60	6054450	H	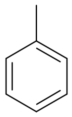	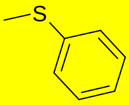	O	O	7.7	1.1	6.8	50%	50%	1.0
61	6347471	H	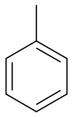	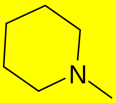	O	O	124.5	1500.0	0.1	0%	0%	-
62	6191422	H	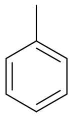	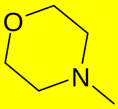	O	O	197.0	345.0	0.6	0%	0%	-

Groupe E

PTF- N°	Chembridge ID	Chemical group (position) R1	R2	R3	Q1	Q2	IC50 (µM) WT	G119S	R_{IC50}	Mortality at 300 µM Slab	SR	R_{m300}
54	5174640	H	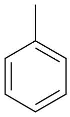	H	O	O	1394.0	737.0	1.9	100%	100%	1.0
63	5909147	H	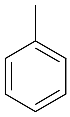	H	S	O	151.0	98.0	1.5	0%	0%	-
64	5809918	H	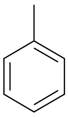	H	N	O	56.0	49.5	1.1	0%	0%	-
65	7171019	H	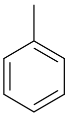	H	N-$C(CH_3)_3$	O	37.0	22.0	1.7	0%	0%	-

Groupe F

PTF- N°	Chembridge ID	Chemical group (position) R1	R2	R3	Q1	Q2	IC50 (µM) WT	G119S	R_{IC50}	Mortality at 300 µM Slab	SR	R_{m300}
66	5975904	H	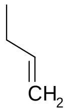	CH_3	O	S	91.6	51.0	2.0	63%	100%	1.6
67	5555858	H	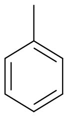	H	O	S	9.1	11.2	0.8	0%	0%	-
68	5810426	H	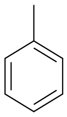	CH_3	O	S	4.0	5.0	0.8	89%	95%	1.1
69	5910400	H	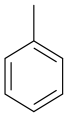	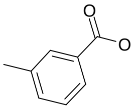	O	S	43.9	4.6	10.0	0%	0%	-
70	6325670	H	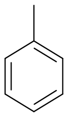	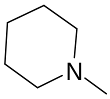	O	S	18.0	6.2	2.9	0%	0%	-
71	6393518	H	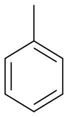	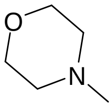	O	S	4495.0	2475.0	1.8	0%	0%	-

Control AChE inhibitors

Inhibitor	Structure	IC50 (µM)			Mortality at 300 µM		
		WT	G119S	R_{IC50}	Slab	SR	R_{m300}
Tacrine	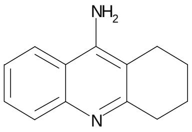	7.4	1150.0	0.01	85%	95%	3.0
Propoxur	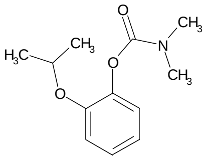	0.3	26500.0	10^{-5}	100%	0%	1.9
Chlorpyrifos-oxon	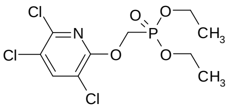	0.01	8.6	0.002	100%	100%	3.4
Paraoxon	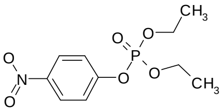	0.17	20.3	0.008	100%	100%	2
Dichlorvos	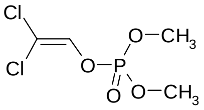	0.45	2.0	0.2	100%	100%	0.7
