# Supplementary material for: Novel AChE Inhibitors for Sustainable Insecticide Resistance Management
Source: PLoS One. 2012 Oct 8;7(10):e47125. doi: 10.1371/journal.pone.0047125 (PMC3466212; doi:10.1371/journal.pone.0047125)
Supplement: Table S2 — Activities of PTFs on G119S, F290V and F331W OP-insensitive AChE1. (PDF) [file pone.0047125.s004.pdf]

Table S2. Activities of PTFs on G119S, F290V and F331W OP-insensitive AChE1.

PTF[a]	IC_{50} (µM)[b]		R_{IC50}	IC_{50} (µM)[b]	R_{IC50}	IC_{50} (µM)[b]	R_{IC50}
	WT	G119S	(WT/G119S)	F290V	(WT/F290V)	F331W	(WT/F331W)
3	29.4	6.2	5	12.4	2	17.8	2
10	592.0	81.4	7	85.9	7	88.2	7
20	330.0	27.5	12	14.5	23	8.3	40
25	442.0	28.6	15	39.3	12	31.9	14
29	118.0	33.5	4	37.4	3	11.2	11
39	74.3	7.6	10	13.9	5	8.7	9

[a] numbers refer to Table S1

[b] IC_{50} values were determined from regression analysis of log-concentrations versus percentage inhibitions. R_{IC50} = IC_{50} WT / IC_{50} G119S, F290V or F331W
