# Supplementary material for: Novel AChE Inhibitors for Sustainable Insecticide Resistance Management
Source: PLoS One. 2012 Oct 8;7(10):e47125. doi: 10.1371/journal.pone.0047125 (PMC3466212; doi:10.1371/journal.pone.0047125)
Supplement: Table S3 — Dissociation constant (K ic ) of the enzyme-inhibitor complex. (PDF) [file pone.0047125.s005.pdf]

Table S3. Dissociation constants (K*ic*) of enzyme-inhibitor complexes.

PTF [a]	K*ic* (µM) [b]		R_{Kic}
	WT	G119S	(WT/G119S)
3	126.6 ± 2.8	21.8 ± 1.9	5.8
10	88.2 ± 4.2	64.5 ± 1.8	1.4
20	56.1 ± 1.8	5.6 ± 0.1	10.0
25	131.3 ± 3.1	24.9 ± 0.6	5.3
29	17.1 ± 6.8	8.9 ± 2.7	1.4
39	130.2 ± 9.2	41.9 ± 2.3	3.1
Tacrine [c]	2.7 ± 0.3	4.4 ± 0.1	0.6

[a] numbers refer to Table S1

[b] data were measured from five independent replicates.

[c] competitive inhibitor reference
